# Removal of artificial sweeteners and their effects on microbial communities in sequencing batch reactors

**DOI:** 10.1038/s41598-018-21564-x

**Published:** 2018-02-21

**Authors:** Shaoli Li, Jinju Geng, Gang Wu, Xingsheng Gao, Yingying Fu, Hongqiang Ren

**Affiliations:** State Key Laboratory of Pollution Control and Resource Reuse, School of the Environment, Nanjing University, Nanjing, 210023 P.R. China

## Abstract

Concern is growing over contamination of the environment with artificial sweeteners (ASWs) because of their widespread existence in wastewater treatment plants (WWTPs). To evaluate ASWs removal and the effect on activated sludge, acesulfame (ACE), sucralose (SUC), cyclamate (CYC) and saccharin (SAC) were introduced individually or in mixture to sequencing batch reactors (SBRs) in environmentally relevant concentrations (100 ppb) for 100 days. Comparisons between ACE removal in a full-scale WWTP and in lab-scale SBRs were conducted. Results showed that CYC and SAC were completely removed, whereas SUC was persistent. However, ACE removal in lab-scale SBRs was significantly greater than in the full-scale WWTP. In SBRs, chemical oxygen demand (COD), ammonia nitrogen (NH_4_^+^-N) and total nitrogen (TN) removal appeared unchanged after adding ASWs (*p* > 0.05). Adenosine triphosphate (ATP) concentrations and triphenyl tetrazolium chloride-dehydrogenase activity (TTC-DHA) declined significantly (*p < *0.05). The mixed ASWs had more evident effects than the individual ASWs. Microbial community analyses revealed that Proteobacteria decreased obviously, while Bacteroidetes, Chloroflexi and Actinobacteria were enriched with the addition of ASWs. Redundancy Analysis (RDA) indicated ACE had a greater impact on activated sludge than the other ASWs.

## Introduction

In recent years, artificial sweeteners (ASWs) have been consumed in substantial quantities in food, beverages, pharmaceuticals and personal care products (PPCPs) and animal feed^1^. They provide no or negligible energy and thus are ingredients of dietary products. These anthropogenic and xenobiotic compounds are high production volume chemicals. With the property of persistence and the highest known concentrations among trace contaminants, they are listed as emerging environmental contaminants^2^.

The widespread occurrence of ASWs such as acesulfame (ACE), sucralose (SUC), cyclamate (CYC) and saccharin (SAC) has been found in wastewater, surface water, groundwater and drinking water systems^3–6^. These four ASWs were found to appear in the aquatic environment at much higher concentrations than most PPCPs and other wastewater-specific anthropogenic organic chemicals^2,7–9^. They have been detected in Europe^10^, the United States^11,12^, Canada^13–15^, Germany^16,17^, and Asia^18–20^. They are usually detected in a few tens ng·L^−1^ to a few tens μg·L^−1^ as the highest known concentration among trace contaminants. For instance, concentrations of the investigated ASWs ranged from 50 ng·L^−1^ to 120 μg·L^−1^ in Tianjin, China^1^. Wastewater treatment plants (WWTPs) are considered to be the main’hotspots’for the release of the four ASWs in the environment^21^.

In most instances, WWTPs present the first treatment opportunity for removing micropollutants and preventing significant environmental exposure^22^. Through investigations in full-scale WWTPs, researchers found CYC and SAC are usually degraded by more than 90% during wastewater treatment; ACE and SUC pass through WWTPs mainly unchanged^2^; thus both can be proposed as tracers of anthropogenic activity^7,13,14^. Castronovo *et al*.^23^ recently found that a maximum median ACE removal of 97% was achieved in three WWTPs using conventional activated sludge treatment with denitrification and nitrification. Falås *et al*.^24^ also observed a significant removal of ACE of up to 80% in bench-scale activated sludge SBRs at a hydraulic retention time (HRT) of 12 h and a solids retention time (SRT) of 10 d. There is a conflict about ACE removal. So, it makes sense to trace the ASWs removal in biological treatment processes, especially ACE.

Biological treatments at WWTPs rely on activated sludge for organics and nitrogen removal. It is essential to illuminate the impact of the anthropogenic compounds on microbial activity and structure community of activated sludge. Denaturing gradient gel electrophoresis (DGGE) and clone library analysis revealed that the microbial community diversity changed with the addition of 250 ppb of tetracycline^25^. Kraigher *et al*.^26^ suggested there was a consistent shift on the bacterial community structure in the bioreactors supplied with pharmaceuticals residues at 50 μg·L^−1^. Pasquini *et al*.^27^ reported that SUC could inhibit the chemical oxygen demand (COD) decrease and even increased the biomass by 20% at 1 g·L^−1^. Tran *et al*.^28^ found that there was a relationship between nitrification and co-metabolic degradation of the target ASWs, especially ACE and SUC. Castronovo *et al*.^23^ reported that neither was ACE removal enhanced in reactors with increased nitrification rate nor did the initial ammonium concentration or the inhibition of ammonium monooxygenase (AMO) effect the degradation rate. However, very few studies have focused on the impact of ASWs on microbial activity and community structure while adding ASWs to the bioreactors. It is necessary to evaluate the association between microbial change and ASWs removal in bioreactors.

In the present study, the interaction between the four target ASWs (ACE, CYC, SAC and SUC) with environmentally relevant concentrations (100 ppb) and microorganisms in activated sludge were investigated. The purpose of this study is: (1) to compare ASWs removal in a full-scale WWTP and in lab-scale SBRs, (2) to investigate changes of reactor performance and microbial activity in activated sludge with addition of the target ASWs, and (3) to determine the effect of ASWs on microbial community characteristics in activated sludge. The study contributes to the further understanding of ASWs removal and their effects on microbial communities in the wastewater treatment process.

## Results and Discussion

### ASWs removal

#### ASWs removal in the full-scale WWTP

**Figure 1:**
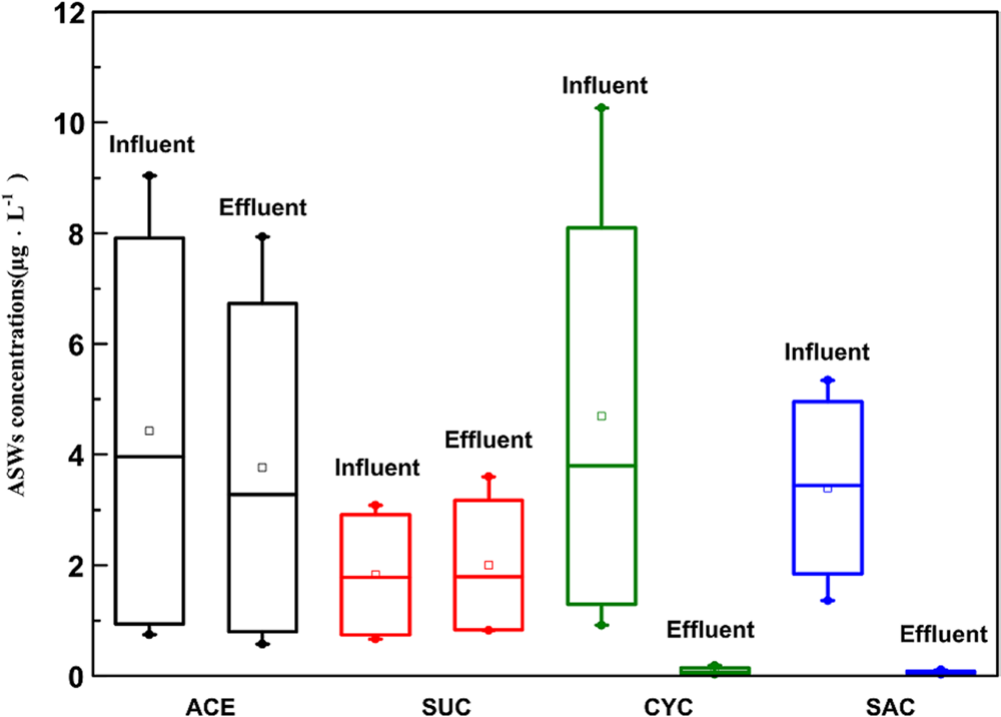
ASWs concentrations in influent and effluent in the full-scale WWTP. The concentration axes have different scales. (The top and bottom of each box represent the 75^th^ and 25^th^ percentiles, respectively; the top and bottom of each whisker represent 90^th^ and 10^th^ percentiles, respectively; the line inside the box represents 50^th^ percentile; the small square represents mean value, and the small circle represents the max and min value).

Figure 1 shows the concentrations of target ASWs in the influent and effluent from the full-scale WWTP. The concentrations of the investigated ASWs in influent decreased generally in the order of CYC > ACE > SAC > SUC, ranging from 0.66 µg·L^−1^ to 10.26 µg·L^−1^. The removal of ASWs are 15.60 ± 6.15% (ACE), −10.78 ± 11.07% (SUC), 96.84 ± 2.47% (CYC), 97.26 ± 3.24% (SAC).The level of ASWs concentrations is similar to previous research reporting a wide range of wastewater concentrations at a few ng·L^−1^ to µg·L^−1 ^^1,12^. CYC and SAC were easily degraded and completely removed in the effluent. The removal of ACE and SUC were both lower than 20%, indicating both of them are persistent. Even SUC was been found negative removal. The lower removal of SUC may be attributed to its physiochemical properties. Chlorine modification makes SUC molecules resistant to biodegradation^29^. Brorström-Lundén *et al*.^29^ suggested that poor removal of the substance may indicate that a fraction of the incoming load was conjugated or complexed and the conjugates (or complexes) were re-transformed back to the mother compound.

#### ASWs removal in lab-scale SBRs

The removal of ASWs in the lab-scale SBRs ranged from 19.86% to 99.83% (Table 1). In general, ASWs concentrations in effluent were found to decrease during the first 20 days compared to those in influent and then stabilized (shown in Figure S1). ACE, SAC and CYC in R_6_ showed the highest removal with 99.92 ± 0.02%, 99.76 ± 3.37% and 99.92 ± 0.01%, respectively. SUC removal was much higher with a maximum of 33.90 ± 0.32%. There is little difference about ASWs removal between individuals and the mixture (*p* > 0.05).

**Table 1:** ASWs removal (%) in different reactors at steady state.

SBRs	ACE	SUC	CYC	SAC
R_2_	98.86 ± 0.03			
R_3_		19.86 ± 3.27		
R_4_			99.86 ± 0.08	
R_5_				99.93 ± 0.04
R_6_	99.92 ± 0.02	33.90 ± 0.32	99.76 ± 3.37	99.92 ± 0.01

By comparison, in the full-scale WWTP (Fig. 1) and lab-scale SBRs (Table 1), both CYC and SAC are easily degradable. This phenomenon is also comparable with those reported in earlier literature^12^. The higher removal of SUC has been found in lab-scale SBRs, reaching the maximum value of 33.90 ± 0.32%. It should be expected that the breakdown of SUC may be slow and incomplete under realistic WWTP operational conditions^30^.

Regarding ACE removal, there was an obvious difference between the full-scale WWTP and lab-scale SBRs. In the lab testing, ACE was almost degraded, while in the full-scale WWTP, the removal rate of ACE was 15.60 ± 6.15%. The compound has been long regarded as persistent to biodegrade with activated sludge^3^, which is incompatible with the result in the study. In a previous study, it showed the removal of ACE was lower than 20% in full-scale WWTPs^28^.

#### Batch tests

The removal of ASWs in activated sludge mostly contributes to hydrolysis, volatilization, sorption and biodegradation. In order to evaluate the fate of ACE in activated sludge specifically, short term batch experiments were performed with both activated sludge (sludge A from the full-scale WWTP, sludge B from lab-scale SBRs) to determine the contribution of hydrolysis, volatilization, sorption and biodegradation.

**Figure 2:**
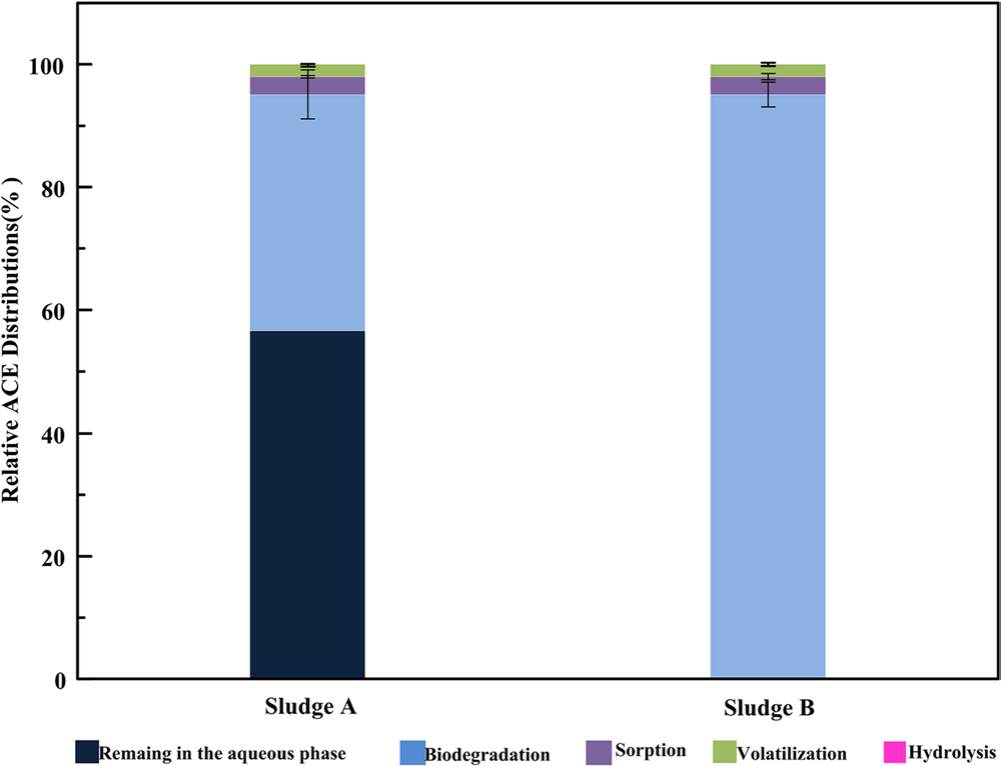
Relative ACE distributions during sludge A and sludge B.

Figure 2 shows the relative ACE distribution during sludge A and sludge B. ASWs removal in different sludge samples is shown in Table S1. In sludge A, approximately 42.92 ± 2.43% of ACE was removed during the aquatic phase. By contrast, ACE was completely degraded in sludge B. All reactors were opaque, so photodegradation was negligible. In the batch tests, we found no hydrolysis occurred during the testing period. Volatilization appeared to account for 2.01%-4.67% of ACE removal in both sludge samples; and the absence of volatilization was in agreement with low Henry’s Law constants^3^. ACE removal due to volatilization and hydrolysis can be ignored. Approximately 2.91%-6.76% of ACE was removed from sorption of sludge. The phenomenon is parallel to low solid-water distribution coefficients (K_d_), which is usually used to evaluate sorption potential. The results indicated that biodegradation played the biggest role in removing ACE. In sludge A, the removal of ACE at 38 ± 1.47% accounted for biodegradation, whereas the biodegradation removal of ACE was 95 ± 2.31% in sludge B. In Figure S1, we discovered that removal of ACE gradually increased with the time of exposure to ACE. It might be that the growth and activity of bacteria contributed to ACE degradation.

#### Reactor performance

The performance of the bioreactors was assessed while adding target ASWs. Figure S2 describes COD removal during the SBRs operation time. The introduction of target ASWs whether individually or in mixtures (*p* > 0.05) did not significantly affect COD removal. In six reactors, the average COD removal ranged from 97.71 ± 2.04% to 98.81 ± 3.48%, suggesting that environmentally relative concentrations of the target ASWs had little effect on COD removal.

Figure S3 and Figure S4 show ammonia nitrogen (NH_4_^+^-N) removal and total nitrogen (TN) removal during the SBRs operation time. The operation conditions of the SBRs hardly fluctuated since the first SRT, so we monitored NH_4_^+^-N removal and TN removal when the target ASWs were added to the SBRs since the first 20 days. There was no significant impact on NH_4_^+^-N removal and TN removal whether they were introduced individually or in mixture compared to the control (*p* > 0.05). Results indicated that the ASWs with environmentally relative concentrations had little effect on NH_4_^+^-N removal and TN removal in the reactors.

These data suggest that there is functional redundancy in the activated sludge microbial community^22^. The sum of bacteria was sufficient for organic matter removal. Therefore, they were not affected by the target ASWs with environmental concentrations. Other studies have come to similar conclusions. For example, Zhang *et al*.^31^ reported SBRs fed with synthetic wastewater with and without antibiotics in environmental concentrations showed similar organic matter biodegradation. Matos *et al*.^32^ also reported that 50 ppb antibiotic had little effect on COD removal and nitrification activity.

### The impact of ASWs on the microbial activity

#### The impact of ASWs on ATP concentrations and live bacteria ratios

**Figure 3:**
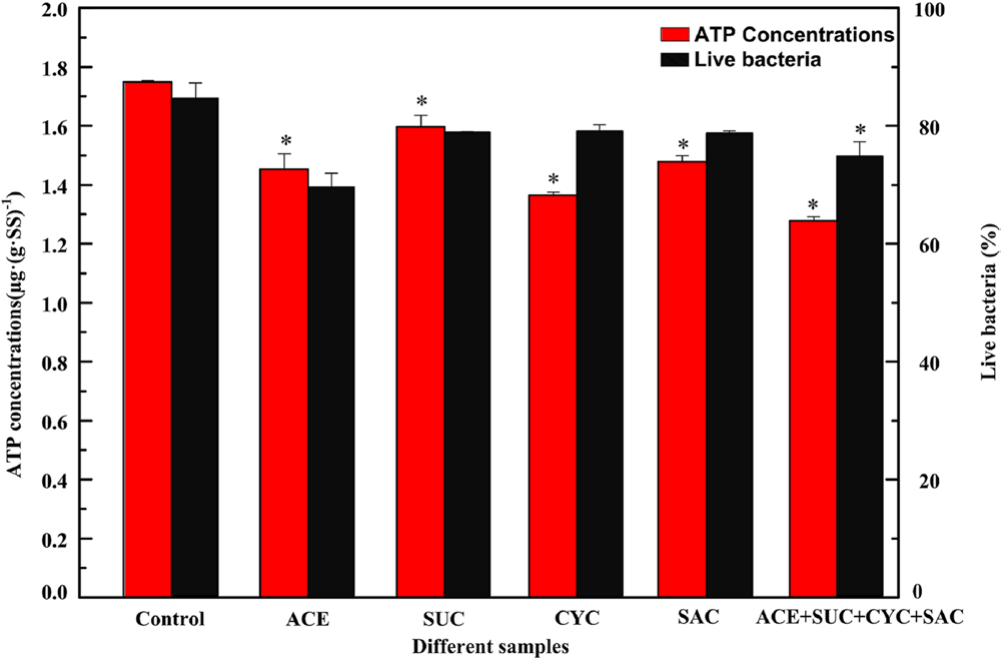
Variations of ATP concentrations and live bacteria ratios in different samples.

Figure 3 describes changes of ATP contents and live bacteria ratios in different SBRs during the steady stage. ATP is essential for microorganism energy storage and is an indicator of microbial growth and metabolism^33^. Dalzell *et al*.^34^ proved that ATP bioassay had been shown to be an effective technique in revealing the effect of pollutants on microorganisms. The ATP concentration was 1.75 ± 0.01 µg·(g·SS)^−1^ in the control. ATP contents decreased with the addition of the target ASWs compared to the control (*p < *0.05). In addition, mixed ASWs (1.28 ± 0.01 µg·(g·SS)^−1^) showed a more obvious decrease than any individual ASW, suggesting that the mixed ASWs may have a synergistic effect on microbial activity. Mu *et al*.^35^ put forward that lower ATP content would indicate weak activity or even the death of microorganisms. So it is likely that ASWs could cause a negative effect on microbial activity in activated sludge.

Live bacteria ratios can reveal membrane integrity and microbial activity when pollutants are dosed in activated sludge^36^. Compared to the control, the ratios of live bacteria reduced when treated with ASWs. Moreover, the addition of ACE and the ASW mixture decreased the ratio significantly (*p < *0.05). The results indicated that ASWs, especially ACE may cause an increased microbial mortality. So, it is likely that environmentally relative concentrations of ASWs may affect microbial mortality and microbial activity. Wang and Gunsch^22^ also found that live bacteria ratios decreased ranging from 16% to 10% with additions of 1 and 10 µM of ketoprofen, naproxen, carbamazepine and gemfibrozil, indicating that four commonly occurring pharmaceutically active compounds affected microbial activity.

#### The impact of ASWs on TTC-DHA

Dehydrogenase is the first enzyme on the metabolites and is a necessary enzyme in the process of microbial degradation of organic matter to gain energy by dehydrogenation. To a great extent, dehydrogenase activity measurement can be used for the determination of bacterial growth and metabolism^37^.

**Figure 4:**
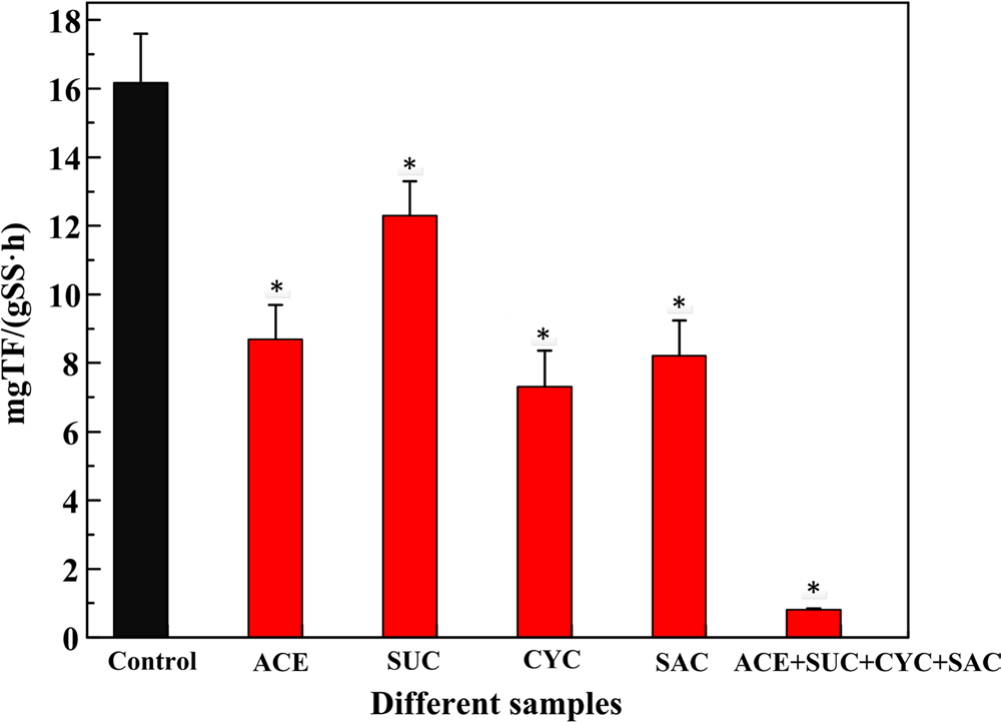
TTC-DHA of microorganism in different samples.

Figure 4 describes the TTC-DHA of activated sludge microorganisms in different SBRs during the steady stage. TTC-DHA of the activated sludge with the target ASWs decreased obviously compared to the control (*p < *0.05). In addition, the addition of mixed ASWs (0.816 ± 0.028 mg·SS^−1^) had a more obvious decrease than the addition of individual ASWs. Muter *et al*.^38^ confirmed that TTC-DHA is ubiquitous in all intact viable microbial cells and DHA is dependent on the presence of nutrients. So it is possible that ASWs could inhibit the activity of bacteria, even its growth. This finding is also supported by the results of ATP concentrations and live bacteria ratios. It has been published that the mechanism of the sulfa antibiotics is interfered by folic acid, which would influence DHA^39^. The target ASWs are sulfonamides except for SUC, so it may influence the synthesis of folic acid and then result in the decline of DHA.

### The impact of ASWs on microbial community characteristics

#### The impact of ASWs on microbial community at phylum level

**Figure 5:**
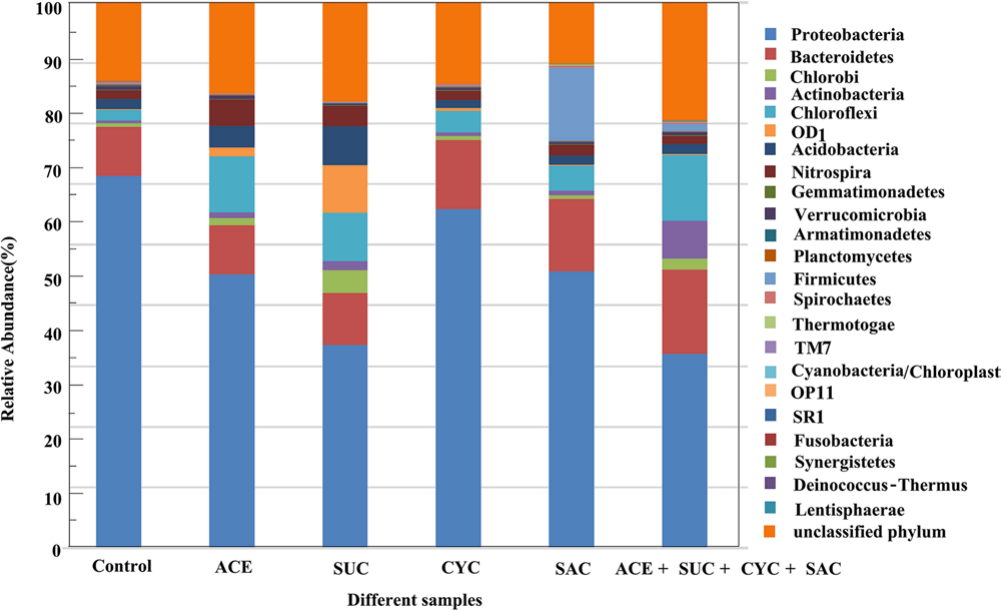
Abundances of different phyla in each activated sludge sample at the steady state.

Figure 5 shows the distribution of 23 dominant bacteria at the phylum level in activated sludge at the steady state. It is evident that Proteobacteria was the predominant phylum in all analyzed samples. In the control test, the percentages of Proteobacteria were 68.17%. After adding the four target ASWs, Proteobacteria abundances were ranging 62.05% to 35.61%. Proteobacteria includes a very high level of bacterial metabolic diversity during the wastewater treatment process^40,41^. The decrease in its abundance indicated that Proteobacteria was found to be vulnerable to the target ASWs load.

Within Proteobacteria phylum, Betaproteobacteria (29.76–14.22%) was the dominant class in all the samples, followed by Alphaproteobacteria (13.59–7.68%), Gammaproteobacteria (19.47–4.95%), Deltaproteobacteria (1.52–3.09%) with a final Epsilonproteobacteria abundance ranging from 0 to 0.20% (Figure S5). The abundance of Gammaproteobacteria obviously decreased under the ASWs addition and set a minimum value in the mixed ASWs. Gammaproteobacteria is an important class of Proteobacteria and can instruct weak flocculation properties^42^.

After the addition of the four ASWs, some special but not abundant bacteria could adapt to the changed environment and gradual increase, such as Bacteroidetes, Chloroflexi, and Actinobacteria. The change indicated that these phyla probably had a stronger tolerance for ASWs. Bacteroidetes are heterotrophic microorganisms and the abundance of the phylum can increase with organic contaminants such amoxicillin^43^. The phylum Chloroflexi constitutes a specialized group of filamentous bacteria only active under aerobic conditions consuming primarily carbohydrates^44^. Kraigher *et al*.^26^ also reported that Chloroflexi increased distinctly after exposure to ibuprofen, naproxen, ketoprofen, diclofenac and clofibric acid in activated sludge. The Actinobacteria abundance increased from 0.58% in control to 6.91% with addition of the mixed ASWs. Actinobacteria are filamentous bacteria and usually have a stronger tolerance for micropollutants such as diesel oil, phenol, polycyclic aromatic hydrocarbons^45^ and sulfonamides^46^. Collado *et al*.^46^ found higher levels of Actinobacteria in biofilms, which had been exposed to higher levels of antibiotics in a Mediterranean river.

#### The impact of ASWs on microbial community at genus level

**Figure 6:**
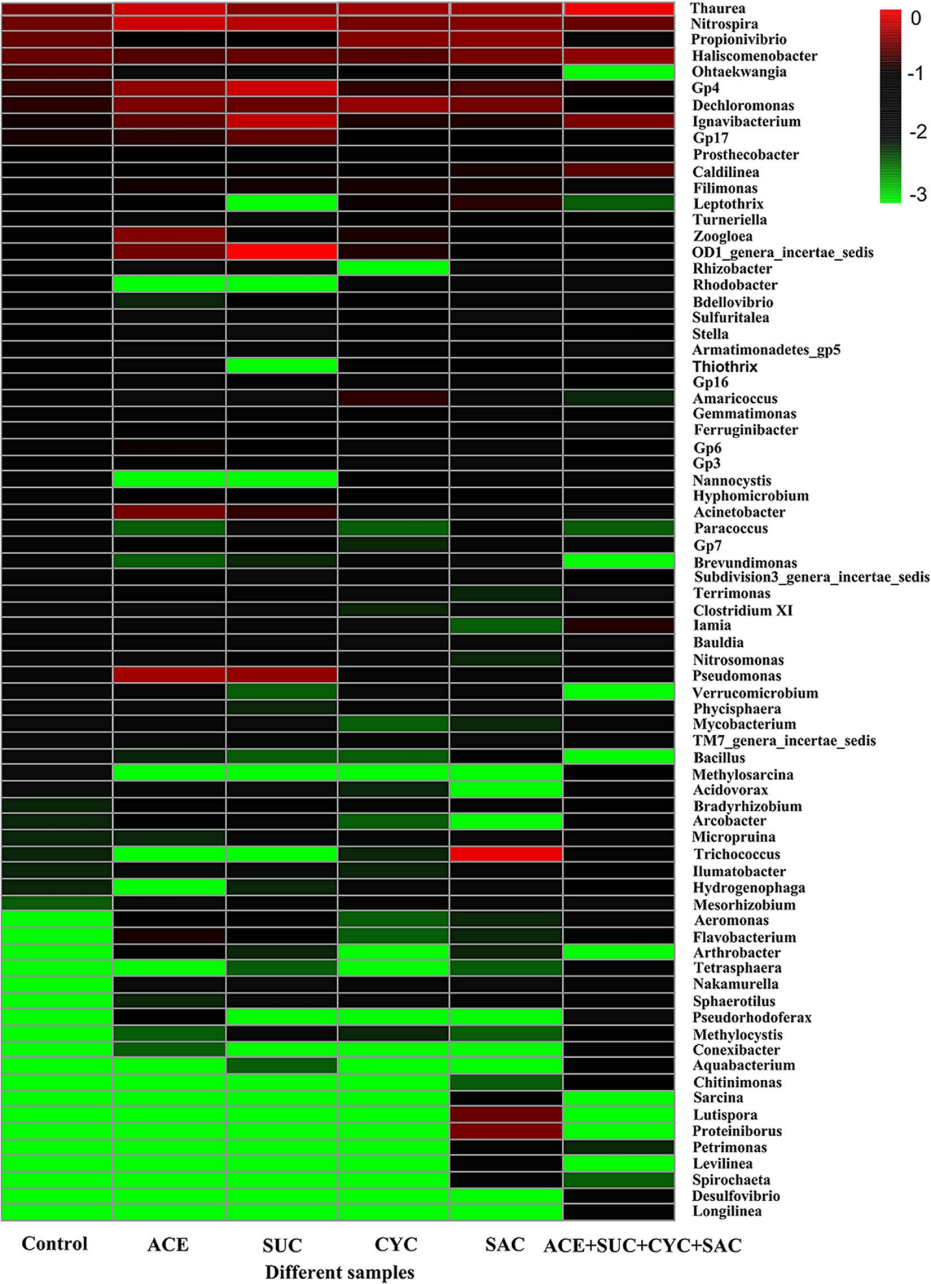
Heatmap of species abundance at the genus level (abundance > 0.1%) in each activated sludge.

Figure 6 shows the heatmap of species abundance at the genus level (abundance >0.1%) in each activated sludge. It is evident that *Thauera*, the second most abundant betaproteobacterial group, has increased from 1.87% (control) to 7.41% (mixture). *Thauera* is a denitrifying genus of Gram-negative bacteria in the wastewater treatment processes and can also be an abundant pollutant degrading members of reactor communities^47^. *Thauera* has been proved to degrade aromatic compounds, such as toluene, benzaldehyde and benzoate under both aerobic and denitrifying conditions. The percentages of *Ignavibacterium* have kept the increasing trendy from 0.54% (control) to 1.87% (mixture). The increase of the genus could contribute to the degradation of lincomycin^48^ and sulfamethoxazole^49^. Li *et al*.^48^ found that *Ignavibacterium* may degrade lincomycin in a sequencing batch biofilm reactor. Wang *et al*.^49^ also found *Ignavibacterium* contributed to the degradation of antibiotic sulfamethoxazole. The chemical structure of ASWs and sulfamethoxazole is similar, which might imply that *Ignavibacterium* could contribute to the degradation of ASWs. The percentages of *Propionivibrio* have kept the decreasing trendy from 1.42% (control) to 0.10% (mixture). The results indicated that *Propionivibrio* may be vulnerable to the addition of ASWs. *Zoogloea* belongs to the Proteobacteria phylum and showed an abundance of 1.98% in the reactor dosed with ACE, compared to 0.23% in the control. *Zoogloea* is a basic functional organism^50^, which is known to have the ability to block toxic compounds from using their exocellular matrix^51^. Augmentation of the abundance of *Zoogloea* indicated ACE may cause toxicity for activated sludge.

As for *Nitrosomonas* and *Nitrospira*, There is no significant difference between the six tests. *Nitrosomonas* is a significant ammonium oxidizing bacteria in activated sludge. Chemolithoautotrophic nitrite oxidizers of the genus *Nitrospira* are monophyletic and play a key role in nitrite oxidation during biological wastewater treatment. These data suggest neither the individual components, nor the combination of the four ASWs had an impact on nitrification. The result was consistent with NH_4_^+^-N removal (Figure S3) and TN removal (Figure S4) in activated sludge samples.

#### Correlation of microbial activity and community structure

**Figure 7:**
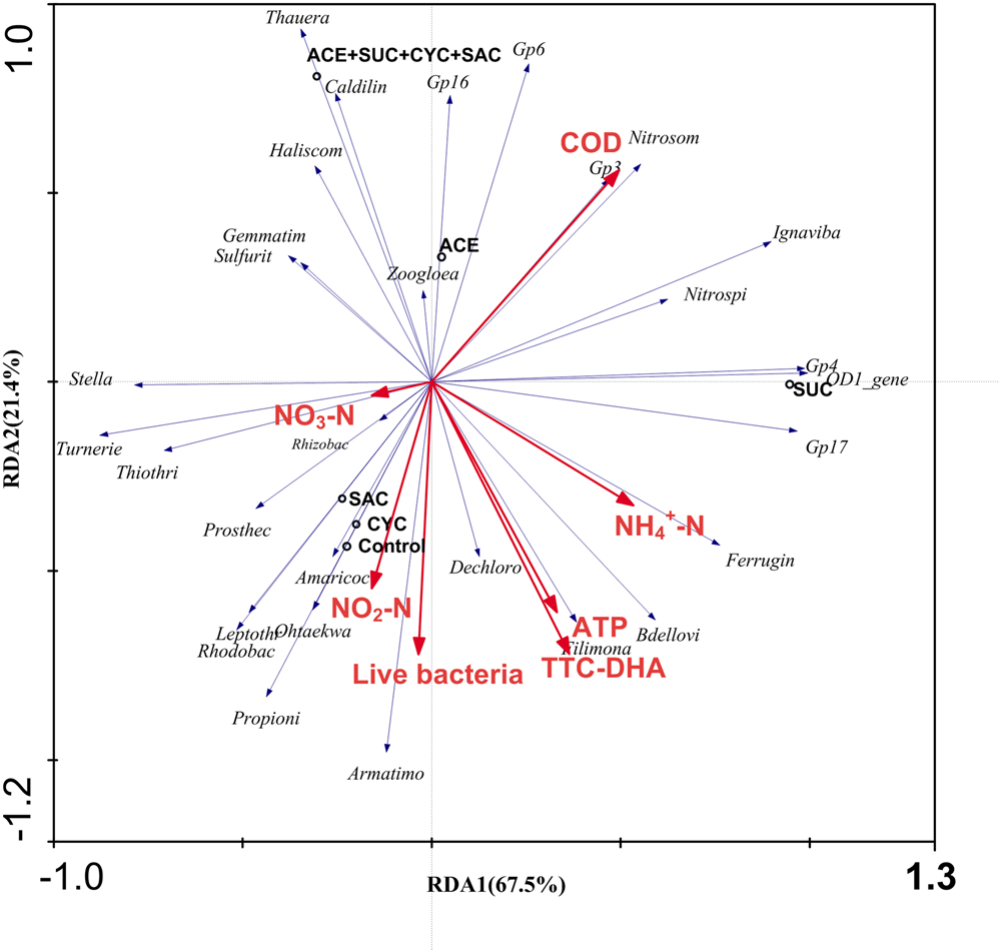
RDA of microbial activity and microbial community in relation to effluent quality.

To investigate the relationship between microbial activity and microbial community in relation to effluent quality, RDA was performed and the results are shown in Fig. 7. It was found that there was an obvious difference between the control and experimental tests. It was observed that ACE and SUC had greater impact on microorganism compared to CYC and SAC. ACE and mixed ASWs affected the microbial activity and community structure similarly, which indicated ACE may have a greater influence on activated sludge than the other ASWs. Figure 7 shows that ATP, TTC-DHA and live bacteria ratios displayed a similar change and were closely correlated to *Filimonas*, *Propionivibrio*, and *Dechloromonas*, all of which are essential players for the removal of micropollutants. NH_4_^+^-N removal and TN removal are related to *Nitrosomonas* and *Nitrospira*.

## Conclusion

This study investigated the interaction between ASWs and microorganisms in activated sludge. ACE, CYC, SAC were completely removed in SBRs whereas SUC was partly degraded by activated sludge in the lab testing. ACE removal was enhanced by biodegradation in lab-scale SBRs. The presence of ASWs did inhibit microbial activity and microbial community structure. The mixed ASWs had more evident effects than the individual ASW. RDA revealed ACE had a greater impact on activated sludge than the other ASWs. Further research regarding actual wastewater is needed to evaluate the impact of ASWs exposure on activated sludge. In addition, the factors about the ASWs degradation rate and degradation mechanism need to be researched in depth.

## Materials and Methods

### Reagents

Acesulfame-K, sucralose, cyclamate, saccharin (> 98% in purity), ammonium acetate and the ion pair reagent TRIS were purchased from Sigma-Aldrich (St. Louis, MO, USA). HPLC-grade methanol and HPLC-grade acetonitrile were supplied by Merck (Darmstadt, Germany). Other chemicals were analytically graded and obtained from Nanjing Chemical Reagent Factory, China. Milli-Q water, with a resistivity of at least 18.2MΩ·cm, was produced from a Millipore purification system (Billerica, CA, USA).

### Samples detection in the full-scale WWTP

Wastewater samples were extracted to detect removal of the four ASWs in a full-scale WWTP located in Nanjing, China. The WWTP treats both municipal wastewater and industrial wastewater. The capacity of the WWTPs is 40000m^3^/d. HRT and SRT are 11.61 h and 11.3 d, respectively. Physico-chemical properties (COD, NH_4_^+^-N and TN and pH) during the process are shown in Table S2. Raw wastewater samples were preserved in darkness at 4 °C until analysis. Samples were filtered using 0.45-μm cellulose nitrate membrane filters. The target ASWs were extracted by a solid phase extraction (SPE) method with CNW Poly-Sery PWAX cartridges (CNW Technologies GmbH, Düsseldorf, Germany) adopted from Gan *et al*.^52^.

### Lab-scale SBRs setup and operation

In this study, lab-scale SBRs were used to simulate bioreactors for evaluating the impact of the target ASWs on the microbial activity and microbial community in activated sludge. Aerobic activated sludge culture was sustained in a 4 L fill and draw reactor with a daily feeding of 300 mg·L^−1^ COD of sodium acetate and 20 mg·L^−1^ NH_4_^+^-N of ammonia chloride. The reactor was 12 cm in diameter, 60 cm in total height and cylindrical in shape. Air was supplied with air diffusers connected to an external air compressor to control the dissolved oxygen (DO) concentration at 4–6 mg·L^−1^. The reactors were operated at room temperature (20 ± 5 °C). SRT was set to 20 d by applying a daily manual purge and HRT was set of 20 h. Each cycle lasted for 12 h, repeated over time in the typical phase sequence: 10 min feeding, 550 min aeration, 150 min settling and 10 min withdraw.

The SBRs were initially inoculated with seed sludge collected from the above WWTP. Before the addition of ASWs, the SBRs were subjected to a startup phase lasting 20 days to make the reactors steady. The mixed liquor suspended solids (MLSS) concentrations in the six reactors were approximately 3000 mg·L^−1^. In addition to sodium acetate and ammonia chloride, microbial growth was also supported with macro and the micro nutrients: KH_2_PO_4_ (21.9 mg·L^−1^), MgSO_4_·7H_2_O (25 mg·L^−1^), CaCl_2_·2H_2_O (30 mg·L^−1^), FeCl_3_·6H_2_O (0.9 mg·L^−1^), CoCl_2_·2H_2_O (0.09 mg·L^−1^), ZnSO_4_·7H_2_O (0.072 mg·L^−1^), Na_2_MoO_4_·2H_2_O (0.036 mg·L^−1^), MnCl_2_·4H_2_O (0.072 mg·L^−1^), KI (0.108 mg·L^−1^), CuSO_4_·5H_2_O (0.018 mg·L^−1^), EDTA-Na (6 mg·L^−1^), H_3_BO_3_ (0.09 mg·L^−1^). The pH was maintained at 7.5–7.8 adjusted by NaHCO_3_ solution.

In this experiment, R_1_ was the control experiment without ASWs addition. R_2_–R_6_ were set with addition of ACE, SUC, CYC and SAC with an environmentally relevant concentration (100 μg·L^−1^) into the synthetic wastewater (Table S3). After adaptation for thrice the SRT, the bioreactors can be recognized as stable^31^. Fifteen mL samples were collected from each reactor every two days for COD removal, NH_4_^+^-N removal and TN removal. ASWs removal was measured for each reactor every seven days. The sludge samples of each reactor were collected during the stable stage (100^th^) for analysis of ATP concentrations, live bacteria ratios, TTC-DHA and microbial community structure.

### Batch tests

In order to analyze ACE distributions in sludge A and sludge B, batch tests were performed in 250 mL Erlenmeyer flasks. Sludge A was taken the aerobic tank of the above full-scale WWTP, and sludge B was collected during the stable stage (100^th^) of lab-scale SBRs. Different activated sludge, ACE and same synthetic wastewater were added the Erlenmeyer flasks. A series of batch experiments are shown in Table S4. The suspended solids concentration was approximately 3000 mg·L^−1^. The flasks were run simultaneously at 20 ± 5 °C for 96 h. All flasks were protected from light to avoid possible photolysis. The removal routes for micropollutants in the activated sludge process are considered to be biodegradation (B), adsorption (A), volatilization (V), and hydrolysis (H). R1 represented all removal routes occurred. In R2, biodegradation was excluded by completely inactivated sludge, which entailed adding sodium azide (NaN_3_) to a final concentration of 1 g·L^−1^. R3 was used to investigate the volatilization and hydrolysis of ACE. R4 accounted for hydrolysis for the elimination of ACE. Samples of the slurry were taken from the batch reactors at the following times: 0, 0.25, 0.5, 1, 2, 4, 8, 12, 24, 36, 48, 96 h. All the tests were duplicate and analyzed at least three times. The data are shown in Table S1.

### Analytical methods

COD, NH_4_^+^-N, TN and MLSS were determined according to Standard Methods for the Examination of Water and Wastewater^53^. DO concentration and pH values were measured using an oxygen meter (SG6, METTLER TOLEDO Inc., USA) and pH meter (FE20, METTLER TOLEDOInc., USA).

ATP is the marker of metabolic activity of bacterial cell and was detected according to Velten, *et al*.^54^ TTC-DHA was detected by spectrophotometry according to Burdock, *et al*.^37^ Live bacteria ratios were detected with LIVE/DEAD BacLight kit (Invitrogen Molecular Probes, USA), which consists of propidium iodide (PI) and SYTO9^36^. The live cells were determined by SYTO9, and PI was used for assessing the dead cells. Each sample from activated sludge (0.1 mL) at the steady state was mixed with the 1.5 µL PI and 1.5 µL SYTO9. After 15 minutes dying, samples were placed on glass slides and observed using a fluorescence microscope (Zeiss, Imager.A1). Twenty random fields were chosen and observed, and live bacteria ratios were calculated by quantifying the areas of images with the image processing software Image J (National Institutes of Health, America).

After pretreatment by SPE, the final ASWs extracted samples were transferred to a 1.5 mL amber vial and stored at 4 °C until analysis. The extracted samples were directly injected into the Ultra Performance Liquid Chromatography-MS Spectrometry system (Waters Xevo TQ-S UPLC-MS system, USA). The LC-MS equipped with an electrospray ionization (ESI) interface operated in negative ionization multiple-reaction monitoring (MRM) mode. Detailed information on the parameters for MRM acquisition can be found in Table S5. The negative ionization mode was set at −2 kV capillary voltage. Nitrogen (with a purity of 99.9%) was used as desolvation gas, with a manipulating temperature of 300 °C. Chromatographic separation was performed with an Acquity UPLC BEH C18 column (2.1 × 50 mm, 1.7 um) at 30 °C in gradient elution mode. The mobile phase was composed of water (A) and acetonitrile (B), both containing 5 mM ammonium acetate and 1 mM TRIS. Gradient elution was carried out at a flow rate of 0.1 mL·min^−1^, and the mobile phase gradient was ramped linearly from 5% to 75% B in 3 min, returned to 5% within 1 min, and the system was allowed to equilibrate for 2 min before the next injection. An injection volume of 20 µL was used for all samples. All samples were analyzed twice.

The linearity of the response was studied using external calibration. Eight-point calibration curves ranging from 0.05 to 1000 µg·mL^−1^ were constructed. The instrumental detection limit (IDL) and method detection limit (MDL) were estimated from the injection of the lowest point of calibration. The IDL and MDL were calculated as 3 and 10 times of the signal-to-noise ratio, respectively. As can be seen in Table S6, satisfactory recovery ranged from 89.91 to 93.73% and the relative standard deviations (RSD, %) were all below 6%. Method precision and method accuracy were determined by six repeated injections of the same water sample during the same day (repeatability) and six injections on five different days (reproducibility). The repeatability and reproducibility of the method for ASWs were 2.7–4.5% and 7.2–11.3%, respectively. When the values were measured below detection limits, we considered them as 50% of MDLs.

### Microbial community analyses

#### Sampling and nucleic acid extraction

The impact of the target ASWs on microbial community structure was monitored by sampling in the reactors at steady state. First, the mixed liquor samples were centrifuged (8000 rpm, 5 min) using the centrifuge (5810 R, Eppendorf) to remove the supernatant for extracting DNA. The method of extraction was the Fast DNA™ Spin Kit for Soil (MP Biomedicals, Santa Ana, CA, USA). The concentration and quality of DNA were determined by NanoDrop 2000 UV-Vis spectrophotometry (Thermo Scientific,Wilmington, DE, USA).

#### 16 S rRNA gene cloning and sequencing

Dominant community members of the activated sludge in SBRs were identified by cloning and sequencing of 16 S rRNA genes. DNA polymerase was used for the amplification of 16 S rRNA genes using PCR Cloning Kit according to Zhang *et al*.^31^ The purified products were sent for sequencing using the Illumina high-throughput sequencing platform (Miseq, Illumina Inc., USA).

### Data analysis

Statistical analyses were performed using the SPSS statistical package. The results were expressed as mean values ± standard deviation (SD), which is shown in the figures. The significant difference between the control and experimental groups were compared by a one-way analysis of variance (ANOVA), and *p* value < 0.05 was accepted as indicating significance. A * significant difference was marked between the control and experimental groups. The values below MDLs were set at 50% of MDLs. Redundancy analysis (RDA) was performed using Canoco software (Version 4.5).

## Electronic supplementary material

Supplementary Information
