# Author Correction: Removal of artificial sweeteners and their effects on microbial communities in sequencing batch reactors

**DOI:** 10.1038/s41598-018-28588-3

**Published:** 2018-07-03

**Authors:** Shaoli Li, Jinju Geng, Gang Wu, Xingsheng Gao, Yingying Fu, Hongqiang Ren

**Affiliations:** State Key Laboratory of Pollution Control and Resource Reuse, School of the Environment, Nanjing University, Nanjing, 210023 P.R. China

Correction to: *Scientific Reports* 10.1038/s41598-018-21564-x, published online 21 February 2018

This Article contains a typographical error in the Acknowledgements section.

“This study was supported by the National Science Foundation of China (No. 21677071), the Jiangsu Natural Science Foundation (No. BK2016057) and National water pollution control and governance of science and technology major special (2017ZX07202003).”

should read:

“This study was supported by the National Science Foundation of China (No. 21677071), the Jiangsu Natural Science Foundation (No. BK2016474) and National water pollution control and governance of science and technology major special (2017ZX07202003).”

