# Supplementary material for: Removal of artificial sweeteners and their effects on microbial communities in sequencing batch reactors
Source: Sci Rep. 2018 Feb 21;8:3399. doi: 10.1038/s41598-018-21564-x (PMC5821853; doi:10.1038/s41598-018-21564-x)
Supplement: Supplementary file 1 — Supplementary Information [file 41598_2018_21564_MOESM1_ESM.pdf]

Removal of artificial sweeteners and their effects on microbial communities in sequencing batch reactors

Shaoli Li，Jinju Geng*，Gang Wu, Xingsheng Gao,

Yingying Fu, Hongqiang Ren

State Key Laboratory of Pollution Control and Resource Reuse, School of the Environment, Nanjing University, Nanjing 210023, P.R. China.

*Corresponding author: Tel: +86 25 89680360. E-mail: jjgeng@nju.edu.cn

Table S1. ASWs removals (%) in different samples during the sludge A and sludge B

Sludge	Time(h)	B1	B2	B3	B4
Sludge A	0.25	4.55 ±0.32	1.26 ±0.19	3.23 ±0.20	0.03 ±0.20
	2	11.77 ±1.12	2.58 ±0.67	4.18 ±0.16	1.23 ±0.09
	4	12.20 ±0.34	4.96 ±0.34	6.32 ±1.12	1.85 ±0.22
	8	16.09 ±2.56	4.23 ±2.21	5.01 ±0.23	0.60 ±0.05
	12	20.08 ±3.59	5.27 ±0.08	1.69 ±0.89	0.75 ±0.18
	24	36.47 ±4.28	3.65 ±0.23	2.56 ±0.32	0.28 ±0.69
	48	41.34 ±1.02	5.59 ±1.85	1.58 ±0.15	0.03 ±0.26
	96	42.92 ±0.39	7.91 ±0.39	5.00 ±2.30	3.08 ±0.10
Sludge B	0.25	12.56 ±0.59	2.46 ±0.05	3.32 ±0.32	0.98 ±0.02
	2	24.89 ±1.89	4.25 ±0.69	1.83 ±0.04	3.21 ±0.45
	4	43.26 ±0.37	2.15 ±0.47	1.54 ±1.16	5.17 ±0.27
	8	77.59 ±5.23	4.39 ±2.08	7.91 ±0.76	0.55 ±0.06
	12	95.01 ±4.16	6.12 ±0.07	3.98 ±0.78	0.51 ±0.14
	24	102.53 ±2.90	9.45 ±0.13	5.67 ±0.47	0.05 ±0.34
	48	100.25 ±1.02	6.31 ±1.89	3.45 ±0.15	0.09 ±0.62
	96	99.92 ±2.03	6.09 ±0.36	3.18 ±1.32	1.18 ±0.13

Table S2. Physico-chemical properties of wastewater from sample sites

WWTP	Process	COD(mg L^{-1})	NH_4^+-N(mg L^{-1})	TN(mg L^{-1})	pH
Full-scale WWTP	Influent	107	19.9	19.9	7.7
	Anaerobic	53	6.7	11.5	7.1
	Aerobic	31	2	7.2	6.7
	Coagulation	17	1.7	6.9	7.3
	BAF	15	1.8	6.7	7.4
	UV	12	1.2	5.7	ND

ND: Not Detected

Table S3. The outline of artificial sweeteners concentrations conditions in SBRs

Reactor code	Artificial sweeteners concentrations
R_1	0
R_2	ACE-100ppb
R_3	SUC-100ppb
R_4	CYC-100ppb
R_5	SAC-100ppb
R_6	ACE-100ppb+SUC-100ppb+CYC-100ppb+SAC-100ppb

Table S4. Batch Tests Design

Treatment	Reactor	Activated sludge	ACE (100ppb)	NaN_3	aeration	Removal routes
1	R1	+	+	-	+	B+A+V+H
2	R2	+	+	+	+	A+V+H
3	R3	-	+	+	+	V+H
4	R4	-	+	+	-	H

B: Biodegradation; A: Adsorption; V: Volatilization; H: Hydrolysis

Table S5. Parameters for MRM acquisition of target ASs

Compound	Parent (m/z)	Daughter (m/z)	Cone Voltage (V)	Collision Energy (V)
ACE	162	82/78	38	14/18
CYC	178	80	60	28
SAC	182	106/42	55	16/26
SUC	395	359/35	70	10

Table S6. Instrumental and method validation data

Targeted ASs	IDL (ng ·L^{-1})	MDL (ng ·L^{-1})	Recovery Mean ±RSD (%)	Repeatability RSD(%)(n=6)	Reproducibility RSD(%)(n=6)
ACE	10	20	89.91 ± 5.18	3.4	9.6
CYC	10	20	93.17 ± 3.76	2.7	10.4
SAC	10	20	92.16 ± 2.25	4.3	11.3
SUC	50	100	93.73 ± 3.12	4.5	7.2

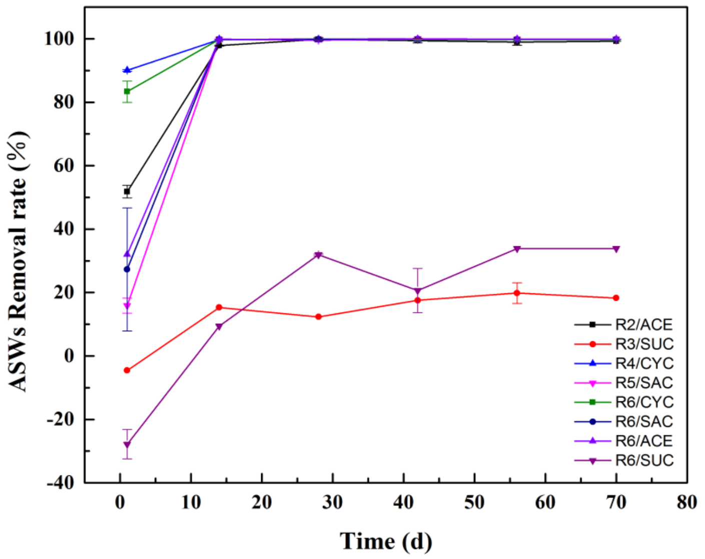

Figure S1. ASWs removal in different samples during SBRs operation

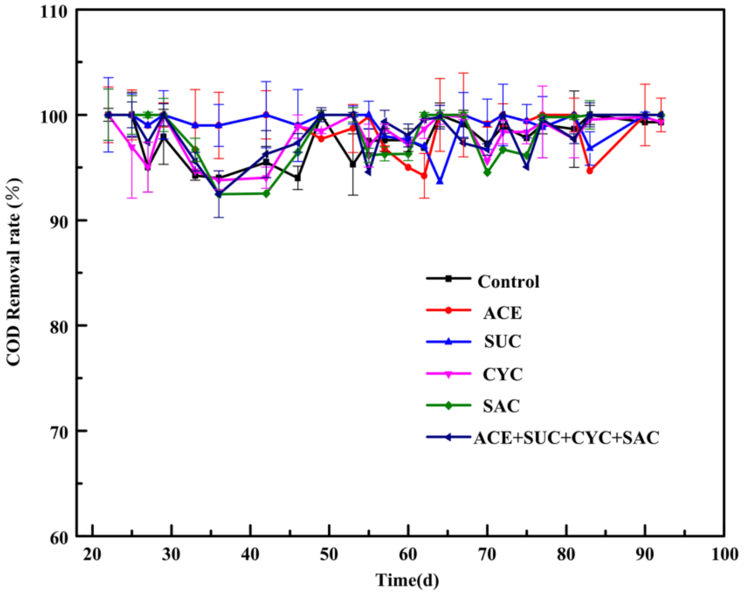

Figure S2. COD removal in different samples during SBRs operation

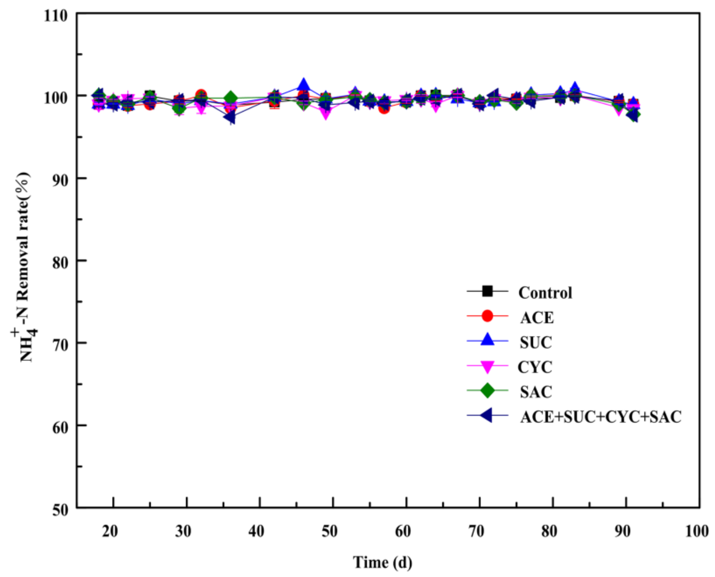

Figure S3. NH_4^+-N removal in different samples during SBRs operation

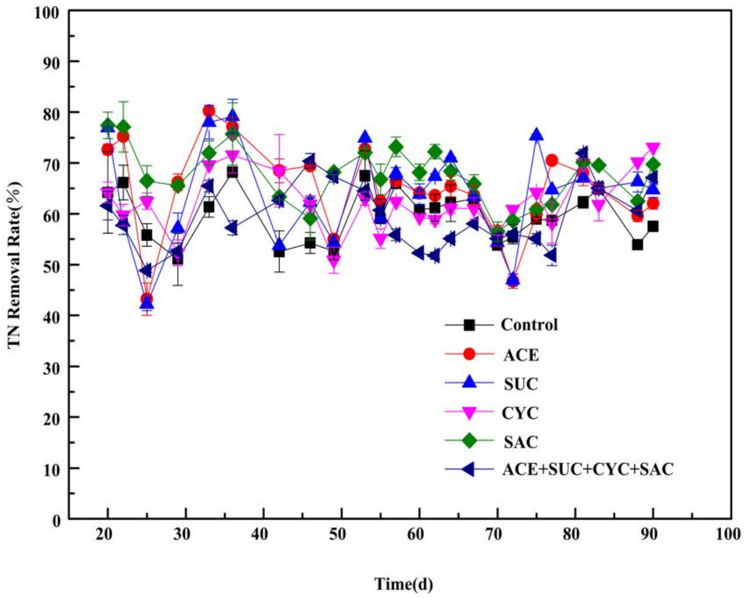

Figure S4. TN removal in different samples during SBRs operation

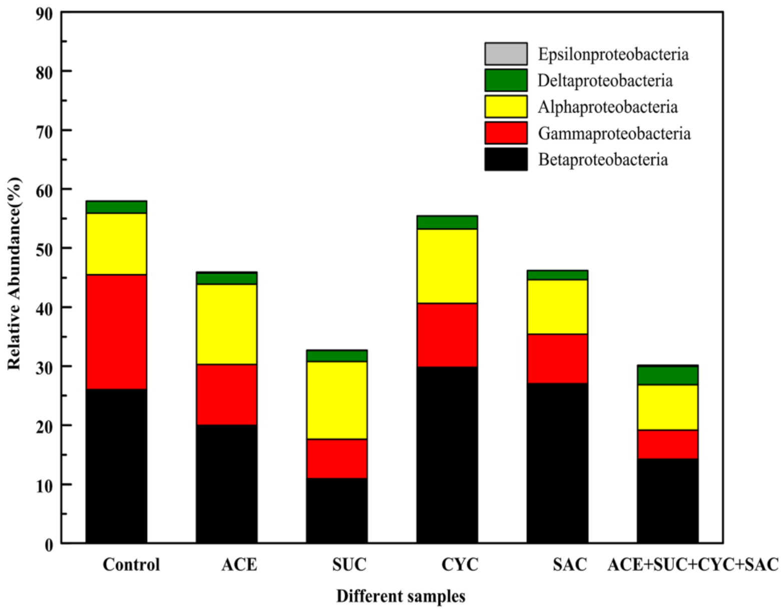

Figure S5. Distribution of Proteobacteria in activated sludge samples
